# Acalculous Cholecystitis Secondary to Hepatitis C Infection

**DOI:** 10.7759/cureus.26484

**Published:** 2022-07-01

**Authors:** Polina Gaisinskaya, Samantha Sugerik, Christopher M Gebara

**Affiliations:** 1 Internal Medicine, Florida Atlantic University, Florida, USA

**Keywords:** hepatitis e, hepatitis d, hepatitis c, hepatitis b, hepatitis a, hepatitis, chronic viral hepatitis, acute viral hepatitis, acalculous cholecystitis

## Abstract

Acute acalculous cholecystitis (AAC) represents gallbladder inflammation without evidence of gallstones. This typically results from gallbladder stasis and/or ischemia, which then causes a local inflammatory response within the wall. The condition is typically multifactorial and seen in critically ill patients, with associated risk factors that include trauma, burns, infections, total parenteral nutrition, and surgery. We present the case of a patient with acute-on-chronic hepatitis C infection leading to AAC.

## Introduction

Only a minority of cases of cholecystitis are acalculous in origin, i.e., about 10%, with an even smaller fraction being associated with hepatitis infection [1]. In more than 50% of cases, an underlying explanation for acalculous inflammation is not found [2]. It is commonly associated with recent surgical procedures, prolonged immobilization, extended starvation periods, and sepsis [2]. While cytomegalovirus, Epstein-Barr virus, hepatitis A, and hepatitis B have been documented in rare cases as causes of acute acalculous cholecystitis (AAC), hepatitis C virus (HCV) is an even rarer cause of AAC. Moreover, the pathophysiology of acalculous cholecystitis as the development of viral hepatitis is not well understood [3]. Possible underlying mechanisms have been reported related to the detergent effect of bile on the epithelium from prolonged stasis, ischemic injury of the gallbladder epithelium, or even immune complex deposition in the vessel wall of the gallbladder, which may then cause necrotizing vasculitis as an extrahepatic complication of chronic hepatitis [4]. Most cases of AAC related to hepatitis are self-limiting [4]. Here, we present the case of a patient with acute-on-chronic HCV infection leading to AAC with uncomplicated recovery not requiring immediate surgical intervention.

This case was previously presented as an abstract poster at the American College of Gastroenterology Annual Scientific Meeting in 2021.

## Case presentation

**Figure 1:**
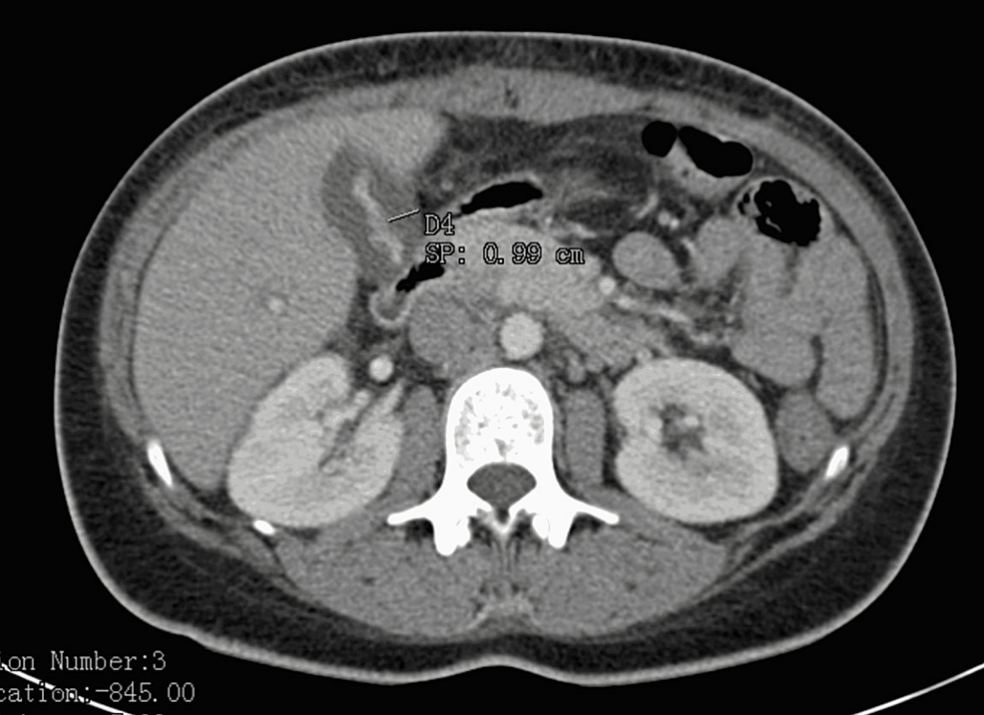
Computerized tomography scan of the patient’s abdomen revealed a 9 mm thickening of the gallbladder wall on admission, consistent with gallbladder inflammation (arrow).

**Figure 2:**
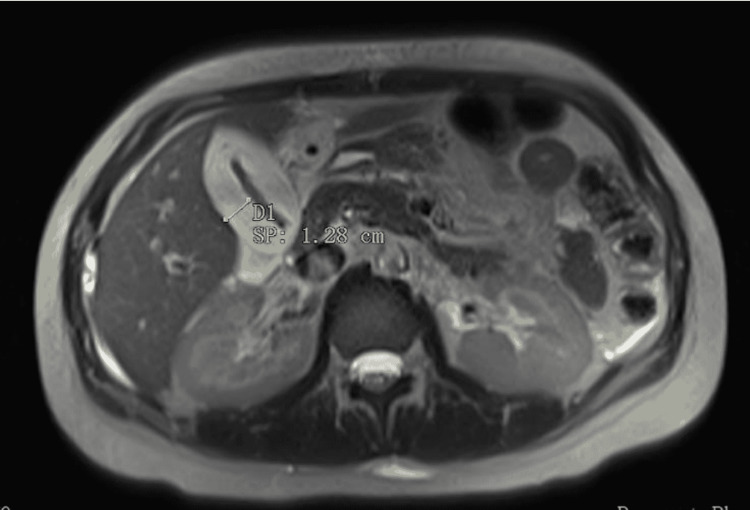
Magnetic resonance cholangiopancreatography of the patient’s abdomen revealed a 1.28 cm thickening of the gallbladder wall (arrow), consistent with gallbladder inflammation.

**Figure 3:**
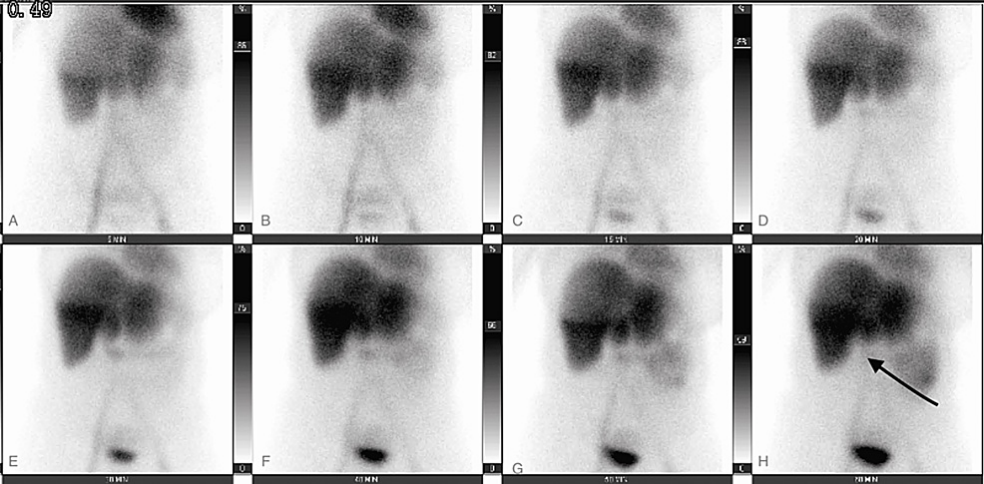
Hepatobiliary iminodiacetic acid scan with failure of visualization of the gallbladder after 60 minutes (Panel H, arrow).

A 27-year-old female with a medical history of intravenous (IV) drug abuse, untreated HCV, endometriosis, and anxiety presented to the emergency department with a four-day history of fever, chills, nausea, vomiting, and right upper quadrant abdominal pain radiating to the epigastric region. She described the pain as stabbing and 7/10 in severity and noted that it was not relieved by ibuprofen or acetaminophen that were taken over the preceding days. It had been two years since she last drank alcohol or injected IV drugs. On physical examination, she had a soft abdomen, mild right upper quadrant tenderness, a negative Murphy sign with normal bowel sounds present, and no visible jaundice. Her labs were significant for total bilirubin at 5.5 mg/dL, direct bilirubin at 3.7 mg/dL, alkaline phosphatase (ALP) at 364 U/L, alanine transaminase (ALT) at 1,739 U/L, aspartate transaminase (AST) at 1,439 U/L, and international normalized ratio (INR) of 1.1. HCV antibodies were positive with an RNA viral load of 104,347. Of note, she was diagnosed in 2018 with HCV and had an undetectable viral load during an elective abdominoplasty performed one month prior to this presentation. She had lost 8 lb since the procedure. Acetaminophen levels, drug screen, autoimmune panel, metabolic panel, and portal venous ultrasound ruled out other etiologies. An abdominal ultrasound revealed an enlarged gallbladder filled with echogenic material thought to be sludge and pericholecystic fluid, with a positive sonographic Murphy sign. Abdominal computerized tomography (CT) scan revealed a normal liver, a contracted gallbladder with 9 mm of wall thickening, and a common bile duct width of 4.6 mm (Figure 1). Subsequent magnetic resonance cholangiopancreatography (MRCP) revealed no evidence of choledocholithiasis and no intra or extrahepatic dilatation but did show marked thickening of the gallbladder wall up to 1.2 cm without stones (Figure 2). A hepatobiliary iminodiacetic acid scan (HIDA) scan was then obtained which failed to visualize the gallbladder (Figure 3, Panel H).

As the patient declined drainage, she was managed conservatively with IV fluids and piperacillin-tazobactam, with gradual improvement of her laboratory abnormalities leading to her discharge after six days with a recommendation for close outpatient follow-up. Unfortunately, the patient was lost to follow-up for further HCV treatment or cholecystectomy.

## Discussion

Only 10% of cholecystitis cases are acalculous in nature [1]. Although the clinical presentation of AAC is similar to that of calculous cholecystitis, acute gallbladder inflammation complicating a severe underlying illness is seen in acalculous cholecystitis [2]. Patients usually, though not always, present critically ill with fever, leukocytosis, or vague abdominal discomfort [3]. Of note, the patient in our case lacked some of these more typical findings. Elevation in the serum total bilirubin and ALP concentrations is not often seen in uncomplicated acute cholecystitis because the obstruction is limited to the gallbladder [1]. In contrast, AAC presents with elevations of both due to obstruction created by the wall edema and gallbladder hypokinesis. Imaging can be helpful in diagnosing a patient with suspicion of AAC; radiologic criteria have been developed for US, CT, and HIDA [4]. US is the modality of choice, and gallbladder wall thickening greater than 4 mm with the presence of sludge is a typical finding associated with AAC. Once diagnosed, the treatment of choice is percutaneous cholecystostomy at presentation [5]. It is thought that the underlying pathogenesis in these cases is due to direct viral invasion of the biliary tract and the gallbladder wall [6,7]. A case report by Omar et al. is the first documentation of AAC developing secondary to acute-on-chronic HCV infection [4]. Similar to our patient, their patient had been diagnosed with HCV infection prior to acute presentation [4]. Their case report cited abdominal US findings of diffuse thickening of the gallbladder wall with lamellated hypoechoic appearance, without gallstones or pericholecystic fluid collection [4]. Similarly, imaging of our patient revealed no gallstones and the main finding of diffuse wall thickening of the gallbladder. In the case reported by Omar et al. and in our case, HCV RNA polymerase chain reaction confirmed flare-up of previously documented HCV infection [4]. Given our patient’s history of untreated HCV, newly risen viral load, and no other predisposing factors to provoke AAC, she was thought to have developed AAC secondary to her HCV status.

## Conclusions

Although AAC is not a common reason for gallbladder inflammation, it is crucial for physicians when working up right upper quadrant pain to consider this diagnosis, as AAC is not often encountered in clinical settings yet is associated with high mortality and morbidity. Although not a common diagnosis in the clinical setting, our case highlights the importance of screening for viral hepatitis when AAC is suspected in the setting of liver function test derangements and no other clear etiology.

## References

[1] Huffman JL, Schenker S (2010). Acute acalculous cholecystitis: a review. Clin Gastroenterol Hepatol.

[2] Loscalzo J, Wiener C, Brown C (2015). Harrison's principles of internal medicine. McGraw-Hill Education.

[3] Barie PS, Eachempati SR (2010). Acute acalculous cholecystitis. Gastroenterol Clin North Am.

[4] Omar A, Osman M, Bonnet G, Ghamri N (2016). Acute acalculous cholecystitis caused by hepatitis C: a rare case report. Int J Surg Case Rep.

[5] Wright WF, Palisoc K, Pinto CN, Lease JA, Baghli S (2020). Hepatitis C virus-associated acalculous cholecystitis and review of the literature. Clin Med Res.

[6] Mohammed RA, Ghadban W, Mohammed O (2012). Acute acalculous cholecystitis induced by acute hepatitis B virus infection. Case Reports Hepatol.

[7] Boninsegna S, Storato S, Riccardi N (2021). Epstein-Barr virus (EBV) acute acalculous cholecystitis in an immunocompromised adult patient: a case report and a literature review of a neglected clinical presentation. J Prev Med Hyg.

